# Early (6 months) results of a pilot prospective study to investigate the efficacy and safety of sirolimus coated balloon angioplasty for dysfunctional arterio-venous fistulas: *MAgicTouch* Intervention Leap for Dialysis Access (MATILDA) Trial

**DOI:** 10.1371/journal.pone.0241321

**Published:** 2020-10-27

**Authors:** Tjun Y. Tang, Shereen X. Y. Soon, Charyl J. Q. Yap, Sze Ling Chan, Ru Yu Tan, Suh Chien Pang, Shaun Q. W. Lee, Hao Yun Yap, Edward T. C. Choke, Chieh Suai Tan, Tze Tec Chong

**Affiliations:** 1 Department of Vascular Surgery, Singapore General Hospital, Singapore, Singapore; 2 Duke NUS Graduate Medical School, Singapore, Singapore; 3 Health Services Research Center, SingHealth, Singapore, Singapore; 4 Department of Renal Medicine, Singapore General Hospital, Singapore, Singapore; 5 Department of General Surgery, Sengkang General Hospital, Singapore, Singapore; Medical University Innsbruck, AUSTRIA

## Abstract

**Background:**

The aim of this pilot study was to evaluate the safety and efficacy of the *MagicTouch*™ sirolimus-coated balloon (SCB) catheter (*Concept Medical Inc*., Tampa, FL, US) on improving the patency of failing arterio-venous fistulas (AVF) with de novo and recurrent stenoses. *MATILDA* reports early outcomes at 3- and 6 months post intervention.

**Methods:**

Single-centre, single-arm prospective pilot study of 33 (18 males; mean age 64.7±11.6 years) end-stage renal failure Asian patients with a dysfunctional AVF, who underwent SCB angioplasty between May 2019-January 2020. All procedures were performed under local anaesthetic without sedation and as day surgery. All patients were prescribed dual antiplatelet therapy for 3 months and followed up with Duplex ultrasound at 3 and 6 months.

**Results:**

47 stenotic target lesions treated and 24/33 (72.7%) patients were for restenosis. Main indications for intervention was low/dropping access flow (21/33; 63.6%) and most common target lesion was in the juxta-anastomosis (19/47; 40.4%). There was 100% technical and procedural success. There were no peri-procedural complications related to the SCB. The target lesion primary patency rates at 3 and 6 months were 46/47 (97.9%) and 29/35 (82.9%) respectively. Circuit access patency rates at 3 and 6 months were 31/33 (93.9%) and 17/25 (68%) respectively. There was one (2.9%) death at 6 months and 4/33 (12.1%) overall to date, all from patients’ underlying co-morbidities.

**Conclusions:**

SCB angioplasty for dysfunctional AVF circuits is a safe and efficacious modality in Asian haemodialysis patients at six months comparable if not better than the paclitaxel data reported to date in the literature.

## Introduction

The recent update to the National Kidney Foundation’s Kidney Disease Outcomes Quality Initiative (KDOQI) Clinical Practice Guideline for Vascular Access introduced an important new concept called the End-Stage Kidney Disease (ESKD) Life-Plan, which maps out vascular access on an individual basis for the lifetime of the patient [1]. Where possible, the creation of an autogenous arteriovenous fistula (AVF) remains the recognized current gold standard for providing haemodialysis. Advantages include improved haemodialysis initiation time, improved dialysis quality, better maintenance of access patency and generally better patient outcomes [2].

Conduit stenosis is the most frequent cause of AVFs becoming dysfunctional and remains a significant cause of morbidity and hospital admission for ESKD patients on haemodialysis [3]. Venous stenosis and scarring are caused by trauma from the surgical access creation when the circuit becomes arterialized and from the repeated percutaneous punctures from subsequent haemodialysis to name a few examples. The KDOQI guidelines recommend treating haemodialysis access stenoses of more than 50% when associated with reduced flow rate and elevated venous pressures [1].

Percutaneous transluminal angioplasty (PTA) with conventional high-pressure balloons are the current standard of care for the treatment of these stenotic lesions but primary access patency rates following conventional balloon angioplasty (CBA) are low–at best achieving a 40–50% 12- month primary patency [4], with high incidence of recurrent stenosis requiring repeat interventions and eventually loss of access and circuit patency [5]. The underlying pathophysiological mechanism is thought to be related to endothelial injury caused by the PTA barotrauma resulting in medial wall hypertrophy and neo-intimal hyperplasia (NIH) [6]. Drug coated balloon (DCB) angioplasty was developed based on a paclitaxel platform to inhibit the NIH effect and reduce the risk of venous restenosis and prolong access patency [7]. It is important to emphasize that the role of drug elution in the treatment of vascular stenosis is not to obtain a good haemodynamic and luminal gain but to preserve a good result obtained during CBA from later restenosis due to NIH and minimise reinterventions and readmissions to hospital for a frail population of patients.

Our group has recently published a systematic review and meta-analysis showing that in patients with dialysis access stenosis, DCB angioplasty was a safe alternative to CBA, offering superior patency rates at both 6 (71.0% vs 49.2%) and 12 months (44.2% vs 20.6%) [8]. This has been borne out in a more recent meta-analysis focusing on AVF per se showing DCB angioplasty to be an effective procedure associated with lower 6- and 12-month target lesion revascularization compared with CBA [9]. However, this was not reflected in the largest RCT to date of DCB vs CBA in AVF with no superior target lesion patency demonstrated at six months and at one and two years follow-up [10]. Furthermore, recent attention has been drawn to a possible increase in late mortality signal [11] and lower amputation free survival [12] in patients receiving DCB treatment with paclitaxel for peripheral arterial disease, although this suggestion has not been demonstrated in the data of DCB within the fistula circuit either at 1 or 2 years [9, 10]. In light of these concerns, attention has turned away recently from paclitaxel-based technologies to sirolimus coated platforms [13]. Sirolimus, like paclitaxel, is a potent antiproliferative agent, which has been found to prevent restenosis in the coronary bed [14] and more recently in the peripheral vasculature [15] but to date has not been studied in AVF circuits.

This study was performed to evaluate sirolimus-coated balloon efficacy and safety using the *MagicTouch™* drug coated balloon catheter (Concept Medical Inc., Tampa, FL, US) on AVF patency with de novo and recurrent stenoses. *MATILDA* reports early clinical outcomes at 3- and 6- months post intervention.

## Materials and methods

### Study design

This is a Singapore General Hospital (SGH) investigator-initiated single investigator (TYT), single arm, non-randomized, prospective pilot study. The trial was carried out under an investigational device exemption (GN27) from the local Health Services Authority (HSA) of Singapore and was designed to test the safety and effectiveness of a sirolimus-coated balloon in haemodialysis fistula–related venous stenoses. Approval was obtained from the local Human Research Ethics Committee (SingHealth Centralised Institutional Review Board reference number: 2019/2561) and the study was carried out in accordance with the *Declaration of Helsinki*. Informed consent was gained from participants. A short patient satisfaction survey was administered at 3 months follow-up.

We hypothesized that the application of SCB to cover the target venous stenotic lesion after successful effacement using a high pressure balloon minimises NIH and improves AVF target lesion patency compared to CBA.

### Study population

33 ESKD patients with failing AVFs on follow-up with the Departments of Vascular Surgery and Renal Medicine at SGH were enrolled between May 2019- January 2020. This allowed for at least 6 months follow-up for 25 patients. Patient demographics, co-morbidities, clinical presentation, history of vascular access, operative details and treatment outcomes were collected prospectively.

The primary inclusion criterion was a native mature AVF in the arm and forearm, which had at least one clinical indicator of dysfunction as defined by the KDOQI [1]. Thrombosed and immature AVFs, those AVFs that had been previously stented, presence of central venous stenosis, and arterio-venous grafts were excluded. There were no restrictions based on gender and race.

### Inclusion criteria

A patient was eligible for inclusion in the study if all the following criteria were fulfilled:

- Patient aged ≥ 21 years and ≤ 90 years
- Native AVF was created more than 2 months prior to the index procedure and had undergone 10 or more haemodialysis sessions utilizing two needles
- Target lesion location had to be located between the anastomosis to the axillary-subclavian vein junction, as defined by insertion of the cephalic vein
- On initial fistulogram, target lesion stenosis had to be > 50% on angiographic assessment and in keeping with the clinical indicator for intervention
- Stenosis had to be < 12cm in length (to allow for potential treatment with one SCB (length 15cm) only
- Stenosis had to be initially treated successfully with a high-pressure plain balloon prior to SCB treatment as defined by:
  - No clinically significant dissection (flow limiting)
  - No extravasation requiring treatment/stenting
  - Residual stenosis ≤30% by angiographic measurement
  - Ability to completely efface the lesion waist using the pre-dilation CBA
- No more than one additional (“nontarget”) lesion in the access circuit that had to be also successfully treated (≤30% residual stenosis) before drug elution. Separate lesion was defined by at least 3cm in distance from the target lesion
- Reference vessel diameters allowed were 5mm– 8mm.

### Exclusion criteria

- Women who were pregnant, lactating, or planning on becoming pregnant during the study
- Subject had more than two lesions in the access circuit
- Subject had a secondary non-target lesion that could not be successfully treated
- Sepsis or active infection
- Asymptomatic target lesions
- A thrombosed access or an access with thrombosis treated ≤30 days prior to the index procedure
- Surgical revision of the access site performed, planned or expected ≤ 3 months before or after the index procedure
- Patients who were taking immunosuppressive therapy or were routinely taking ≥ 15 mg of prednisone per day
- Currently participating in another investigational drug, biologic, or device study involving sirolimus or paclitaxel
- Contraindication to aspirin or clopidogrel usage
- Mental condition rendering the subject unable to understand the nature, scope and possible consequences of the study, or language barrier such that the subject is unable to give informed consent
- Uncooperative attitude or potential for non-compliance with the requirements of the protocol making study participation impractical
- Where final angioplasty treatment requires a stent or drug eluting balloon > 8mm in diameter
- Metastatic cancer or terminal medical condition
- Blood coagulation disorders
- Limited life expectancy (< 12 months)
- Allergy or other known contraindication to iodinated media contrast, heparin or sirolimus

### MagicTouch™ sirolimus coated balloon

The sirolimus-coated balloon MagicTouch™ (Concept Medical Inc., Tampa, FL, US) is coated with sirolimus homogeneously through spray coating. Sirolimus is encapsulated in a protective lipophilic package, allowing diffusion and penetration into the vessel wall during balloon inflation. The total dose of sirolimus corresponds to 1.25 mg/mm^2^ of the surface of the balloon. The sizes of balloons available for this study were 5mm to 8mm in diameter. The 5mm and 6mm sizes were available on an 0.018” wire platform in 150mm balloon length and 150cm shaft length. The 7 and 8mm balloon diameters were available on the 0.035” platform with balloon lengths of 8cm or 15cm and shaft length of 80cm.

### Mustang® high pressure non-compliant balloon

The Mustang® catheter (Boston Scientific, MA, US) is a true non-compliant over-the-wire low profile balloon that is deployed over an 0.035” wire platform with an expansive size matrix, designed for treating vascular stenotic lesions. It has a high burst pressure (24 RBP) aimed for opening more calcified and resistant lesions. It has a low balloon compliance for controlled opening of resistant lesions and allows for safe and precise inflation. There is minimal diameter growth even at burst pressure. Mustang® catheter offers a full-size matrix—available with balloon diameter sizes 3.0, 4.0, 5.0, 6.0, 7.0, 8.0, 9.0, 10.0 and 12.0mm with lengths from 20mm-200mm, and working shaft lengths of 40cm,75cm and 135cm. The balloon is made of Nybex material (proprietary co-extrusion of Nylon–provide strength for lower profile and higher RBPs and Pebax–allows trackability), which eliminates trade-off between pressure and deliverability. The bi-lumen and uncoated shafts are designed for fast deflation and enhanced handling, with a low withdrawal force when it has been inflated and deflated.

### Procedure

All procedures were performed in a hybrid operating theatre by the senior author (TYT), who is a high volume experienced endovascular surgeon. The patient was positioned in the supine position with their fistula arm horizontally laid out and all interventions were performed under local anaesthesia with no sedation and as a day case procedure. The site and initial direction of the access were selected on the basis of the physical examination findings and prior songraphic results. An initial digital subtraction fistulogram was obtained using a short Prelude 6Fr Brite tip sheath (Merit Medical, Utah, US). Stenosis was identified by visible narrowing of the lumen more than 50%; if there were any doubt, orthogonal projection images were acquired. The stenosis was crossed with a hydrophilic 0.035” or 0.018” guidewire (Boston Scientific, Marlborough, MA, USA), with a Berenstein 1 support catheter (Cordis Corporation, Milpitas, CA, USA). The lesion was pre-dilated with a standard high-pressure balloon (Mustang®, Boston Scientific, Marlborough, MA, USA), size and length dictated by the fistulogram findings. The balloon was inflated to a pressure enough to efface the lesion and maintained invariably for 120 seconds. In general, the balloon diameter was chosen to match the calibre of the adjacent normal vessel. If the stenosis persisted along with recoil, these lesions were excluded from drug elution. The SCB (MagicTouch™) was subsequently inserted and inflated for 2 minutes to allow maximal drug transfer to the vessel wall. Balloon length was chosen so that it was 2 cm longer than the area treated during pre-dilatation (1cm overlap proximal & distal) to avoid geographical miss. The SCB was chosen to be 0.5mm– 1mm bigger at burst pressure than the biggest high-pressure balloon used. No post-dilatation was allowed. A post-procedural fistulogram was performed to ensure a good angiographic result. Following vascular sheath removal, haemostasis of the puncture site was achieved using a dissolvable purse-string suture.

Patients were started post-operatively on dual antiplatelet agents for a duration of 3 months with proton pump inhibitor cover. Patients were clinically evaluated at 3- and 6 months post SCB implantation as part of standard AVF care with Duplex ultrasound imaging. If a patient experienced an access problem, such as decreased dialysis flow rates, cannulation difficulty, recirculation and abnormal limb swelling in between follow-up visits, they underwent urgent Duplex scanning and clinical assessment in view for further endovascular intervention.

### Definitions and outcome measures

Standard definitions based on the SIRS guidelines were used [16]. Technical success was defined as the successful inflation of the SCB with < 30% residual angiographic stenosis. Procedural success was defined as technical success with at least one indicator of hemodynamic or clinical success.

The primary efficacy end point was target lesion primary patency (TLPP) at 6 months defined as no need for clinically driven reintervention of the target lesion or access thrombosis and no significant restenosis (lumen diameter < 2.7mm) on Duplex ultrasound [17]. A patient could undergo reintervention on the AVF without TLPP ending provided that the reintervention did not include retreatment of the target lesion. The primary safety end point was freedom from localized or systemic serious adverse events through 30 days that reasonably suggest the involvement of the arteriovenous access circuit. These would include life-threatening events or those resulting in death, requiring hospitalization, resulting in permanent disability, or requiring intervention to prevent permanent impairment; the latter definition included thrombosis. Secondary end points included access circuit primary patency at 6 months. Access circuit patency loss was defined by a development of a stenosis in any region of the AVF circuit requiring intervention. Other secondary endpoints included target lesion primary patency and access circuit primary patency at 3 months, procedural success, access circuit thrombosis, need for open bypass revision surgery, abandonment, number of interventions required to maintain access circuit primary patency at 3 and 6 months and mortality.

### Statistical analysis

Based on the known 6-month primary patency of approximately 40% with CBA, we estimate that 6-month primary patency could improve to 70% following SCB treatment and estimated that the sample size needed was 21 subjects using an alpha risk of 0.05 and a power of 0.8 to demonstrate superiority by a margin of at least 5%. Hence a follow-up of n = 25 at 6 months should suffice.

Baseline variables were summarised with the use of descriptive statistics. Continuous variables were reported as the mean and standard deviation, or median and range, as appropriate, and categorical variables as absolute number and percent. Univariable analysis for continuous and categorical variables were performed using Mann-Whitney U test and Fisher’s exact tests respectively. Patency of intervention was defined as the duration between the index intervention to the time another intervention was required to maintain access patency. The probability of re-intervention over time was calculated using competing risks analysis using the *cmprsk* package in R, as death was a competing risk. Cause-specific sub-distributions were compared across groups using Gray’s test. All analyses were performed in R version 3.5.1 [18].

## Results

33 non-consecutive patients (47 target lesions; 48 SCB) were treated with combination high pressure CBA and SCB inflation over 8 months. There were 18 (54.5%) males and the mean age was 64.7 ±11.6 years. The majority of patients were Chinese (22/33; 66.7%) and 21/33 (63.6%) were diabetic. There were 15 (45.5%) radiocephalic, 10 (30.3%) brachiocephalic and 5 (15.2%) 2-stage brachiobasilic fistulas treated. 24/33 (72.7%) had previously undergone endovascular salvage of their fistula with CBA alone and none had a history of AVF thrombosis. The mean access age at time of dysfunction was 997 (± 901) days. Baseline demographics are shown in Table 1.

**Table 1:** Patient demographics.

	Number of subjects (n = 33)	Percentage (%)
Mean Age, years (±sd)	64.7 ± 11.6	
Mean BMI, kg/m^2^ (±sd)	26.1 ± 5.8	
Gender		
Male	18	54.5
Female	15	45.5
Ethnic Group		
Chinese	22	66.7
Malay	10	30.3
Indian	1	3.0
Smoker	5	15.2
Co-Morbidities (%)		
Hypertension	32	97.0
Hyperlipidemia	30	90.9
Diabetes	21	63.6
Coronary Artery Disease	11	33.3
Asthma	4	12.1
Cerebrovascular Accident	3	9.1
Access Side		
Left	25	75.8
Right	8	24.2
Access Type		
Radiocephalic	15	45.5
Brachiocephalic	10	30.3
Brachiobasilic	5	15.2
Radiobasilic	2	6.1
Ulnarbasilic	1	3.0
Mean Access Age, days (±sd)	996.7 ± 901.0	
Mean Access Flow, ml/min (±sd)	439.2 ± 256.5	
Medical History		
Statin	24	72.7
Antiplatelet	20	60.6
Antidiabetic agents	20	60.6
Beta Blocker	19	57.6
Warfarin	1	3.0

**Fig 1:**
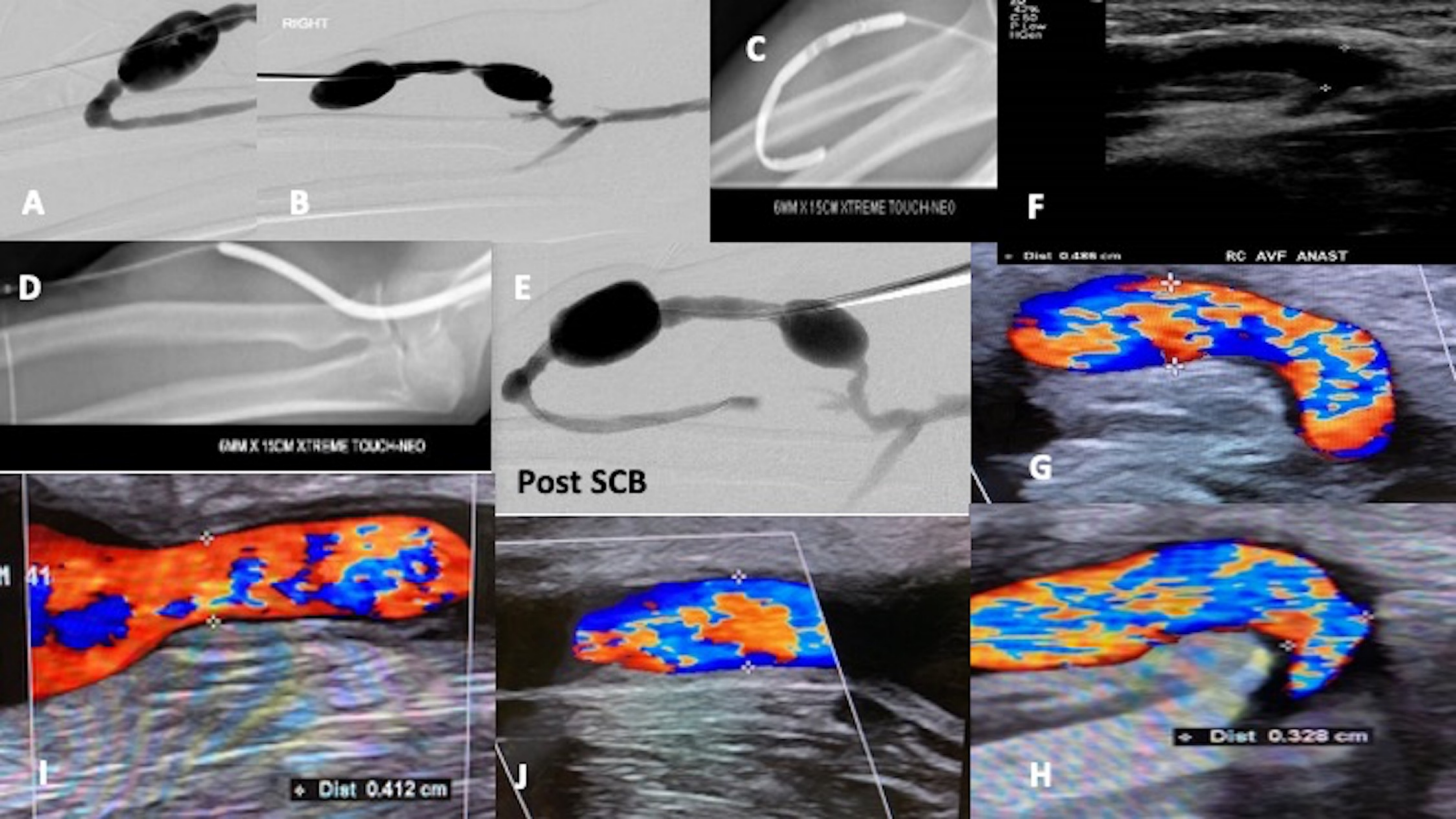
Case-study using sirolimus coated balloon. 74-year-old Malay male with a right mid-forearm fistula of 4.2 years maturity presenting with dropping access flow and having had CBA within 3 months prior **(A)** pre-plasty fistulogram showing a juxta-anastomotic stenosis **(B)** intra-aneurysmal segment stenosis and cephalic outflow vein stenosis located near antecubital fossa **(C) & (D)** use of sirolimus coated balloon drug elution post pre-dilatation with high pressure non-compliant balloons for 2 minutes **(E)** final fistulogram result with good luminal gain of both target lesions **(F)** baseline ultrasound post SCB at juxta-anastomosis (JAS) portion **(G)** Duplex ultrasound at 3 months post intervention at JAS and **(H)** 6 months post treatment at JAS **(I)** inter-aneurysmal segment portion of cephalic vein at 3 months and **(J)** same area at 6 months showing minimal change and access flow. One thing to note is that the NIH effect in the wall of the fistula was less pronounced at 3 and 6 months.

Main indications for intervention were low or dropping access flow in 21/33 (63.6%) and cannulation difficulties in 6/33 (18.2%). Main location of target lesion was in the juxta-anastomosis (defined as within 5cm length from the anastomosis) 19/47 (40.4%). The most common size SCB used was 6mm in 30/47 (62.5%) target lesions. Indications and details regarding intervention are presented in Table 2. Technical and procedural success was 100%. There was no conduit rupture during any of the procedures requiring bailout stenting. See Fig 1 for example images of a case using SCB. There was no puncture site haemorrhage or infection requiring treatment or readmission. Bruising was observed along the circuit track in 4/33 (12.1%) patients, all had resolved at the first follow-up appointment. There were no serious adverse events reported within 30 days that could be attributable to the procedure or SCB.

**Table 2:** Procedural details.

	**Number of subjects (n = 33)**	**Percentage (%)**
**Indication for Intervention**		
Dropping Access Flow	21	63.6
Cannulation Difficulties	6	18.2
High Venous Pressure	4	12.1
Arm Swelling	1	3.0
Recirculation	1	3.0
Recurrent	24	72.7
De novo	9	27.3
	**Number of target lesions (n = 47)**	**Percentage (%)**
Location of target lesion		
Juxta-anastomotic Segment	19	40.4
Proximal Cephalic	9	19.1
Cannulation Zone	7	14.9
Proximal Basilic	5	10.6
Cephalic Arch	4	8.5
Mid Cephalic	3	6.4
De novo	13	27.7
Recurrent	34	72.3
Mean time from prior intervention, days (sd)	298.6 ± 207.6	
Mean stenosis, % (sd)	83.7 ± 9.7	
Mean lesion length, mm (sd)	34.7 ± 19.2	
	**Number of SCBs (n = 48)**	**Percentage (%)**
Balloon diameter (mm)		
5	7	14.6
6	30	62.5
7	4	8.3
8	7	14.6
Mean CBA inflation pressure, atm (±sd)	19.3 ± 4.6	
Mean SCB inflation pressure, atm (±sd)	11.4 ± 2.1	

CBA; Conventional Balloon Angioplasty.

SCB; Sirolimus Coated Balloon.

33 (100%) were available for 3-month follow-up and 25 (75.6) were reviewed at 6 months and overall mean follow-up of 5.45 ± 1.56 months (range 2.30–7.63 months). During that time all used their AVF for haemodialysis and did not require a temporary line. As of May 2020, overall cohort mean time to reintervention was 4.44 ±1.49 months and mean time to TLR was 6.43 ±1.81 months. Further interventions were required in two patients at 3 months (one TLR for recoil, stenosis approximately 70%) and a further 5 patients by 6 months making a total of 8/33 (24.2%) by 6 months (Table 3). All required one intervention to keep access patency. All reinterventions were with CBA only (no further SCB), requiring no stent bail out. Degree of stenosis was less than 30% for lesions that did not require further interventions at 3-months (46/47, 97.9%). No AVFs were lost or abandoned during follow-up and no patient required haemodialysis via a vascular catheter. There were 4/33 (12.1%) deaths during follow-up from unrelated causes (acute myocardial infarction, cerebral vascular accident, 2 cases of ischemic heart disease) at 2.3, 6.5, 7.5 and 8.7 months respectively.

**Table 3:** Procedural outcomes.

**3-months**	**Patent** (%)	**Re-intervened** (%)	**p-value**
Mean Access Flow, ml/min (±sd)	718.4 ± 373.4		
Circuit Access Patency (n = 33)	31 (93.9)	2 (6.1)	
De Novo (n = 9)	8 (88.9)	1 (11.1)	
			0.477
Recurrent (n = 24)	23 (95.8)	1 (4.2)	
Reasons for re-intervention	High venous pressures (1)		
	Dropping access flow (1)		
Target Lesion Primary Patency (n = 47)	46 (97.9)	1 (2.1)	
De Novo (n = 13)	12 (92.3)	1 (7.7)	
			0.277
Recurrent (n = 34)	34 (100.0)	0	
JAS lesion (n = 19)	19 (100.0)	0	
			1.000
Non-JAS lesion (n = 28)	27 (96.4)	1 (3.6)	
Time to target lesion reintervention, months	1.73		
**6-months**	**Patent** (%)	**Re-intervened** (%)	
Circuit Access Patency (n = 25)	17 (68.0)	8 (32.0)	
De Novo (n = 6)	3 (50.0)	3 (50.0)	
			0.344
Recurrent (n = 19)	14 (73.7)	5 (26.3)	
Reasons for re-intervention	AVF thrombosis (3)		
	Dropping access flow (3)		
	High venous pressures (1)		
	Difficulties with cannulation (1)		
Target Lesion Primary Patency (n = 35)	29 (82.9)	6 (17.1)	
De Novo (n = 9)	7 (77.8)	2 (22.2)	
			0.635
Recurrent (n = 26)	22 (84.6)	4 (15.4)	
JAS lesion (n = 13)	10 (76.9)	3 (23.1)	
			0.648
Non-JAS lesion (n = 22)	19 (86.4)	3 (13.6)	
Mean TLR-free duration, months (±sd)	7.17 ± 2.36		
De Novo (n = 9)	6.18 ± 2.40		
			0.145
Recurrent (n = 26)	7.51 ± 2.29		
JAS lesion (n = 13)	6.99 ± 1.78		
			0.731
Non-JAS lesion (n = 22)	7.28 ± 2.68		
Mean time to target lesion reintervention, months (SD)	4.44 ± 1.49		

JAS; Juxta-Anastomotic Stenosis.

TLR; Target Lesion Revascularization.

The TLPP rates at 3 and 6 months were 46/47 (97.9%) and 29/35 (82.9%) respectively. Circuit access patency rates at 3 and 6 months were 32/33 (93.9%) and 17/25 (68%) respectively. The estimated median target lesion revascularization free duration was 7.37 months (IQR 5.89–8.81 months).

After 3 months, 31/32 (96.9%) patients were either extremely satisfied or very satisfied with their treatment. 30/32 (93.8%) patients indicated they would choose this procedure again if given the choice.

### Subgroup analysis

We also looked at whether there were subgroup outcome differences between de novo and recurrent lesions and whether the TLR rate were different if the lesion was located in the juxta-anastomosis or elsewhere.

**Fig 2:**
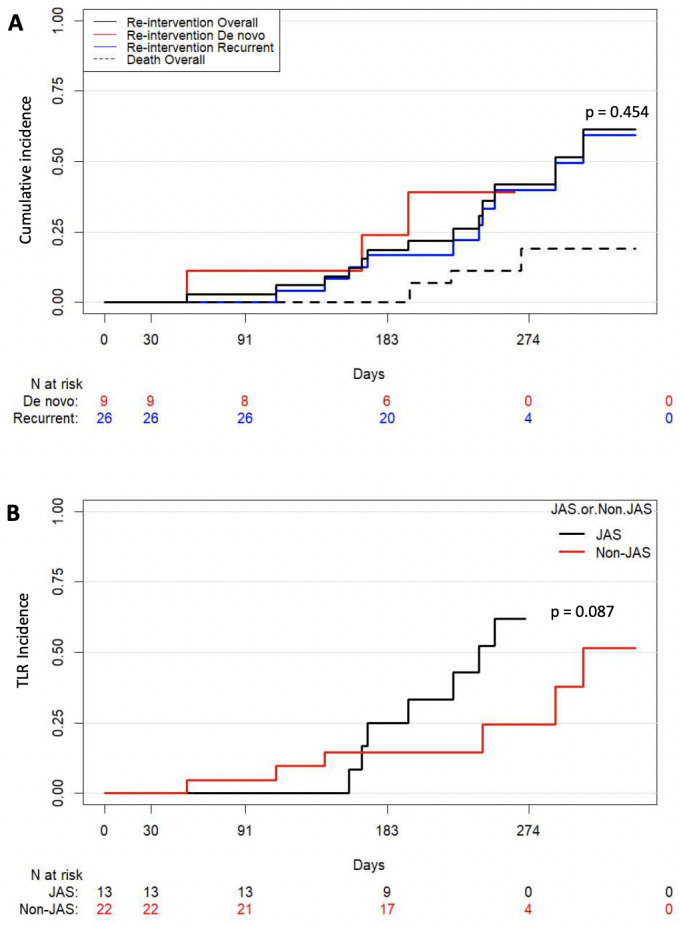
**(A)** Cumulative incidence of TLR for lesions at 6-months (n = 35) and subgroups [De novo lesions (n = 9), Recurrent lesions (n = 26)] and cumulative incidence of deaths at 6months **(B)** Cumulative incidence of TLR for subgroup JAS (n = 13) and non-JAS (n = 22) lesions.

The TLPP rate was higher albeit insignificant for the recurrent compared to the de novo lesions at both 3 months (100% vs 92.3%; p = 0.277) and 6 months (84.6% vs. 77.8%; p = 0.635). The estimated median TLR-free duration of combined CBA/SCB was higher for recurrent vs de novo lesions (7.77 months [IQR 6.57–8.93 months] vs. 6.53 months, IQR 5.53–8.07 months]; p = 0.131) but this did not reach significance. Fig 2A shows competing risk analysis curves for cumulative incidence of TLR for all lesions at 6-months (n = 35) and subgroups (de novo vs recurrent).

The estimated mean TLR- free duration in patients with a juxta-anastomotic stenosis (6.99±1.78 months; 95% CI: 6.02–7.95 months) was not significantly higher than those with non-juxta-anastomotic lesion (7.28±2.68 months; 95% CI: 6.16–8.40 months; *p = 0*.*731*). The TLPP rates in patients with a juxta-anastomotic stenosis were higher but did not have statistical difference compared with a non- juxta-anastomotic stenosis at 3 months (100% vs. 96.4%; *P* = 1.000) but this effect was reversed at 6 months (76.9% vs. 86.4%; *P* = 0.648) albeit insignificant. Fig 2B shows competing analysis curves for cumulative incidence of TLR for JAS and non-JAS lesions.

## Discussion

This is to our knowledge the first study to publish safety and six-month efficacy data on the use of sirolimus coated balloons in the AVF haemodialysis circuit. The main findings demonstrate excellent technical and procedural/clinical success (100%) in combination with a preparatory pre-dilatation high pressure non-compliant balloon (Mustang®, Boston Scientific, Marlborough, MA, USA). Furthermore, it delivered impressive 3- and 6- month TLPP of 93.9% and 82.9% respectively and a median clinical TLR-free duration of 7.4 months. These results are comparable if not superior to the paclitaxel-coated balloon (PCB) TLPP published data for AVF circuits and the six-month data is likely to be better than stated because there are further patients awaiting Duplex follow-up imaging, without any access problems, which have not been included in the analysis. A recent meta-analysis comparing the effectiveness and safety outcomes comparing PCB vs CBA for AVF stenosis, showed that PBA was associated with significantly lower 6-month TLR rate of 22.8% at 6 months compared with 47.4% at the same time interval for CBA, despite the use of more high- pressure balloons in the CBA group [11]. In another meta-analysis [19], the pooled primary patency rate of the PCB group at 6 months was 75.2% but it was noted that the data used for both reviews had statistical heterogeneity. The two largest randomised controlled trials (RCTs) to date comparing PBA vs CBA in AVF are worth a mention only to highlight the TLPP for comparison. The *Lutonix AV Randomised Trial* was the first prospective, global, multicenter (*n* = 23), randomized, controlled trial that compared the efficacy and safety of PCB-assisted angioplasty (*n* = 141) with CBA (*n* = 144) in patients with dysfunctional mature AVFs [11]. The TLPP for the PCB group was 71% compared to 63% for the CBA group and although there was a trend for better patency for PBA, the pre-specified 6-month primary efficacy endpoint was not met. Two-year results have recently been published showing that as time passed, the efficacy of the PCB became more variable [11]. Although fewer interventions were needed to maintain TLP in the PCB group at 9 months this was not reflected at the 12 –or 24 months interval. Recently 12 -month outcomes of the *IN*.*PACT AV Access* study (n = 330) were presented showing an impressive 82.2% and 63.8% TLPP in the PCB arm at 6- and 12 months respectively compared to 59.5% and 43.6% TLPP in the CBA arm [20]. This was the first PCB versus CBA trial to meet its primary effectiveness endpoint and that there was a highly significant advantage in terms of CD-TLR for the PCB treated group. Our high TLPP may also be down to good vessel preparation using a versatile high pressure non-compliant balloon, to prepare the way for the sirolimus to penetrate the fistula wall layers. We allowed a good 120 seconds of high pressure CBA (mean 18 atm) prior to performing drug elution for a further 2 minutes to maximise wall drug transfer. A similar balloon inflation protocol was performed in the Aperto Registry [21] showing excellent 6-months TLPP of 88%, albeit with a PCB device.

In the past year, worldwide use of paclitaxel has dramatically reduced because of the medium term adverse mortality signal raised by the *Katsanos* meta-analysis for the peripheral vasculature [11]. The results based on pooled data of over 4500 patients showed an increased mortality rate at 2 and 5 years, following the application of paclitaxel balloons or stents compared to non-drug devices in the femoro-popliteal artery region. However, this trend has never been shown in the AVF circuit from either the meta-analysis data [9] or from the larger RCTs [10] and registries [21]. If the current trend continues, paclitaxel devices may be ultimately consigned to the past [13] and this will be a setback for progress as many vascular specialists perceive CBA and bare metal stents to be basic technologies with poorer outcomes. The mortality rate of 12.1% (4 deaths) all from cardiovascular-related causes may seem slightly alarming but these are patients with multiple co-morbidities and vascular risk factors. Deaths occurred outside the peri-operative 30 day window and is in keeping with the mortality rate of dialysis dependent patients (12–14% per annum) from the Singapore Renal Registry, which reports that the most common causes of death are cardiac and infection related [15].

In other applications such as in the coronary circulation, sirolimus has shown better long-term safety and efficacy compared to paclitaxel [14]. The drug is generally seen to be better than paclitaxel because of better late lumen loss and reduced restenosis rates [22]. Both sirolimus and paclitaxel can impact endothelial function, but while paclitaxel inhibits cell viability (cytotoxic), sirolimus (or analogs) have a cytostatic mode of action with a greater margin of safety, and additional anti-inflammatory properties [23]. Historically, packaging sirolimus with enough quantity onto a balloon platform that can be transferred effectively to the arterial wall has proven to be challenging because sirolimus has poor bioavailability compared to paclitaxel. Sirolimus in its natural state has slow tissue absorption, necessitating the use of a co-solvent to enhance tissue uptake. Furthermore, sirolimus has a larger size of 43 micrometers compared to paclitaxel, and is, therefore, unable to penetrate through the intimal, medial and adventitial layers of the artery. Novel phospholipid-encapsulated sirolimus nanocarriers coated onto balloon platforms have demonstrated efficient transfer of sirolimus to all layers of the vessel wall in animal models, achieving high tissue concentration of drug for a number of days after the procedure [24]. This has formed the foundation for the use of sirolimus in the peripheral vasculature [25] and one of the noted phenomenon has been the incredible minimal late lumen loss seen in the *Selution Trial* at 2 years, which investigated sirolimus elution to the femoral-popliteal arteries of claudicants [26]. We have also seen this in the AVF circuit in this study with minimal encroachment within the fistula lumen from the NIH effect using Duplex ultrasound at 3 and 6 months post SCB angioplasty and this may be explained by sirolimus being equally distributed in the vessel layers in contrast to paclitaxel, which only accumulates in the adventitia and is believed to play an inferior role in preventing restenosis [23].

The response to drug coated angioplasty may differ among stenotic lesions at different sites, such as the juxta-anastomosis, cephalic arch, or cannulation zones and whether the lesion is recurrent or de novo [27]. Restenotic lesions are usually prone to frequent reinterventions. Irani *et al*. demonstrated that PCB angioplasty offered a greater benefit for restenotic lesions in more mature AVF circuits [28] but the Aperto Registry showed that the potential for restenosis inhibition by paclitaxel may well be greatest in younger fistulas [21]. We have shown from our data with sirolimus that recurrent lesions seem to have a better TLPP at both 3 and 6 months than de novo lesions, albeit insignificant. Also data suggests that juxta-anastomotic lesions seem to respond better than other lesions to paclitaxel elution because the inflammatory response is more pronounced in this region and hence the anti-inflammatory properties of PCB would be an advantage [29] but our SCB results only reflect this trend at 3 months, perhaps due to limited patient numbers and event rates.

### Limitations

Limitations of this study include its small sample size, non-randomized and single arm nature, albeit being a pilot and novel to the existing literature on AVF endovascular salvage. However the nature in which the patients were recruited by the principal investigator (TYT) and broad inclusion criteria reflect a real-life registry of heterogeneous Asian ESKD patients presenting with distal and proximal lesions in their AVF. The lack of comparator group precludes any statement on patency rates in this population using alternative interventions however. Another limitation is the SCB lengths used—we were provided only with 8cm and 15cm length balloons. Ideally, we would have only liked to cover the angioplasty zone with 1 cm overlap proximal and distally but there are cases where local administration of sirolimus was also applied to non-stenotic fistula segments. This resulted in a higher dose of sirolimus applied than required and may cause unnecessary barotrauma to non-stenotic areas.

A strength of the analysis is the completeness of the follow-up data and the prospective nature of the study. Although the therapeutic effect of combined high pressure CBA and SCB angioplasty looks promising, cost-effectiveness needs to be performed in any future RCT as it is likely to be an expensive undertaking. We look forward to reporting one-year patency data. Furthermore the limited follow-up (six months) represents only preliminary data and we intend to report patency and outcome at one and two years.

## Conclusion

SCB angioplasty combined with high pressure CBA vessel preparation for dysfunctional AVF circuits is a safe and efficacious modality in Asian haemodialysis patients at six months comparable if not better than the paclitaxel data reported to date in the literature. Future randomized controlled trials comparing limus- to taxol-based technology for the failing AVF are urgently required.
